# Phenological mismatches and the demography of solitary bees

**DOI:** 10.1098/rspb.2022.1847

**Published:** 2023-01-11

**Authors:** Diego P. Vázquez, Nydia Vitale, Jimena Dorado, Georgina Amico, Erica L. Stevani

**Affiliations:** ^1^ Argentine Institute for Dryland Research, CONICET & National University of Cuyo, Av. Ruiz Leal s/n, 5500 Mendoza, Argentina; ^2^ Freiburg Institute for Advanced Studies, University of Freiburg, Albertstrasse 19, 79104 Freiburg im Breisgau, Germany; ^3^ Faculty of Exact and Natural Sciences, National University of Cuyo, Padre Jorge Contreras 1300, M5502JMA Mendoza, Argentina; ^4^ Centro para la Conservación de la Biodiversidad, Secretaría de Ambiente y Desarrollo Sustentable, Municipalidad de Mendoza, Ituzaingó 1425, 5500 Mendoza, Argentina

**Keywords:** demography, phenological mismatches, plant–pollinator interactions, reproductive success, solitary bees

## Abstract

Species respond idiosyncratically to environmental variation, which may generate phenological mismatches. We assess the consequences of such mismatches for solitary bees. During 9 years, we studied flowering phenology and nesting phenology and demography of five wood-nesting solitary bee species representing a broad gradient of specialization/generalization in the use of floral resources. We found that the reproductive performance and population growth rate of bees tended to be lower with increasing nesting–flowering mismatches, except for the most generalized bee species. Our findings help elucidate the role of phenological mismatches for the demography of wild pollinators, which perform key ecosystem functions and provide important services for humanity. Furthermore, if climate change increases phenological mismatches in this system, we expect negative consequences of climate change for specialist bees.

## 1. Introduction

Phenology—the timing of periodic life-history events—can vary through time in response to fluctuation in environmental conditions [1]. Different organisms respond idiosyncratically to environmental fluctuation, which may lead to phenological mismatches among interacting species [2–8]. Such mismatches may have demographic consequences—what has been termed the match/mismatch hypothesis [2,4,7,9,10]. Proposed originally in the context of fisheries, the match/mismatch hypothesis posits that in seasonal environments, the recruitment of a consumer is determined by the degree of temporal overlap between the consumer's reproductive season and its resources [2,4,7,9–11]. Put in a broader ecological context, this hypothesis predicts that, when they occur, phenological mismatches have stronger effects on specialist than generalist species, as the former should be less plastic in resource use than the latter [7,12–14].

Studies evaluating the match/mismatch hypothesis have assessed whether consumer–resource phenological mismatches resulting from changes in average climatic conditions influence negatively the reproductive success and population abundance of the interacting species [15–21]. These studies have focused on long-term, gradual shifts in the phenologies of interacting species, with little emphasis on the arguably pervasive non-directional, year-to-year variation in phenological matching among interacting organisms, which is also likely to have profound ecological and evolutionary consequences [22]. In addition, to our knowledge no previous studies have evaluated the demographic consequences of phenological mismatches for a set of species with contrasting degrees of specialization.

Here, we attempt to fill the above knowledge gaps. We evaluate the match/mismatch hypothesis based on 9 years of data on flowering phenology and bee nesting events and demography in a dryland ecosystem for a group of five wood-nesting solitary bee species that represent a broad gradient of specialization/generalization in the use of floral resources. Bees are an ideal group to assess this hypothesis, as they depend on floral resources (pollen, nectar and sometimes nest-building materials) throughout their entire life cycle [23], which makes them particularly sensitive to phenological mismatches. Bees also play an essential role as pollinators in natural and agricultural ecosystems [24,25]. Specifically, we hypothesized that bee recruitment would be determined by the degree of temporal overlap between bee nesting and the flowering periods of their main floral resources, and that such phenological mismatches would have stronger effects on specialist than generalist bee species, as the former depend on a narrower array of resources than the latter. Thus, according to this hypothesis we predicted that bee recruitment (i.e. the number of brood cells per nest) and the resulting population growth rate would decrease with increasing phenological mismatch between bee nesting and flowering, and that such effects of mismatches would be stronger for specialist than for generalist bees. We evaluated these predictions by estimating the nesting–flowering phenological mismatch and per-nest brood production of our study bee species. Furthermore, because effects on individual nests do not necessarily have an impact on population demography [26], we also assessed the population-level effect of mismatches on reproductive success and, ultimately, population growth rate ($\bar{\lambda }$) for each study bee species.

## 2. Methods

### (a) Study area and sites

We conducted the study in two 100 m × 200 m sites located at approximately 1240 m above sea level in Villavicencio Nature Reserve (32.54° S, 68.96° W; 32.52° S, 68.94° W), Las Heras Department, Mendoza Province, Argentina. The predominant vegetation is representative of the Monte Desert ecoregion [27,28]. Mean annual rainfall ranges 150–350 mm, concentrated during spring and summer, which combined with high evaporation leads to a permanent water deficit [29].

### (b) Flower abundance

Our estimate of flowering phenology of the plant species present in our study sites is based on weekly estimates of the floral density in fixed plots and transects, conducted during the flowering season of most plant species (September–December). Because data come from several projects running consecutively at the same sites, there are minor differences in the sampling methods used in different years. Thus, during the flowering season, we estimated floral density weekly at fixed quadrats/transects: forty regularly spaced 2 m × 2 m quadrats in 2006, five fixed 50 m × 2 m transect belts in 2007 and four 8 m × 20 m plots plus two 2 m × 50 m transects in the remaining years (2008–2014).

Whenever possible we counted all flowers of each plant species in a plot or transect. When there were too many flowers to count in a plot or transect, we counted the number of flowers in at least ten individuals and then estimated the number of flowers by multiplying the average number of flowers per individual by the number of individuals in the plot or transect. When we could not distinguish flowering individuals (some herbaceous species), we counted all flowers in the plot or transect. We included in the study all flowering plant species assumed to be animal pollinated (excluding for example grass species).

### (c) Bee nesting, bee reproductive success and identification of pollen for larval provision

We used wooden trap nests to study bee nesting phenology and reproductive success. Each trap nest consisted of two wood pieces bound together with paper tape with a longitudinal hole drilled in between them (electronic supplementary material, figure S1). We used this design so that we could open the nests regularly to examine their contents without damaging the brood cells. We used trap nests of 5 mm in internal diameter and 14 cm in tunnel length, 8 mm diameter and 14 cm length, and of 11 mm diameter and 28 cm length; we did not use the latter diameter during the first study year. We placed these trap nests in the field in bundles with eight trap nests of each diameter (24 total) at multiple sampling points in each study site. Trap bundles were attached to shrubs in the first two study years, and to metal poles in the remaining years (electronic supplementary material, figure S1). In each site, we placed trap nests at 30 points separated at least by 20 m from each other (2006 and 2007) or at twelve paired points separated 100 m from each other (2008–2014; electronic supplementary material, figure S1). The number of trap nests per site and year, the trap dimensions and other relevant information of the sampling design are shown in electronic supplementary material, table S1 and figure S1. Although the study design varied slightly between the first two and the remaining seven study years, the number of trap nests of each size per site was similar among years, and so this variation is unlikely to affect our estimate of the number of brood cells per nest, the main reproductive variable used in our study.

Bees included in this study belonged to four genera and five species: the carder bee *Anthidium vigintipunctatum*; the petal-cutting bees *Megachile leucographa* and *M. nigella* (identified as *M. ctenophora* in previous publications in the same study area [30–32]); the wood-borer bee *Trichothurgus laticeps*; and the carpenter bee *Xylocopa atamisquensis*. Other bee species also nested in our trap nests [31,32], but their abundance was too low to allow quantifying the variables needed to address our research questions.

We checked trap nests weekly during the bee breeding season. Once the construction of a nest had ended, we removed the occupied traps from the trap bundles and replaced them by empty traps. During the first three study years, we took to the laboratory all traps with nests for rearing and identification purposes, while during the remaining years we took to the laboratory only traps for which identification was not possible in the field; we removed other nests from the bundles but left them in the field, a few metres away from the original nesting site. For the nests taken to the laboratory, we opened traps to record the number of cells; whenever the nest had more than one brood cell, during the first three study years we extracted one cell for pollen identification, and kept the remaining cells until adult emergence. We identified pollen to the lowest possible taxonomic resolution: species in most cases, genus in a few cases with several species in the same genus, and morphospecies in a few rare pollen grains that we could not identify. We conducted pollen identification by light microscopy using the natural method [33,34], in comparison with a reference collection that included all flowering plant species recorded in our study plots [35]. We kept some emerged adults of each nest for identification and returned the remaining individuals to their site of origin in the field. After the first three study years (i.e. from 2009 onwards), whenever possible, we identified the bee species in the field without taking the nests to the laboratory. We recorded the number of brood cells and their taxonomic identity in all nests.

### (d) Estimation of bee generalization

We defined bee generalization as Shannon's diversity index of the pollen found in all nests of each species in the 3 years in which we conducted pollen studies (2006–2008). To calculate Shannon's diversity index we started from the number of pollen grains of each plant species in each nest, suspending the pollen from each extracted brood cell in an ethanol–water solution, from which we took three samples for pollen quantification under a microscope. We counted 15 microscope fields for each sample, and 45 fields total for each brood cell. With these data, we calculated the proportion of pollen grains of each plant species in each nest, which we used as a standardized measure of plant abundance per nest, so as to remove the effect of the total number of pollen grains counted per nest. We then summed the standardized abundance of each plant species across all nests as a measure of the abundance of that plant species in the population of a bee species. This is the abundance we used to calculate Shannon's diversity index. The sample size for this estimation included 18 nests for *A. vigintipunctatum*, 51 nests for *M. leucographa*, 8 nests for *M. nigella*, 32 nests for *T. laticeps* and 14 nests for *X. atamisquensis*. We obtained similar estimates of bee generalization with a larger dataset, using an additional twelve sites in the same study area used in another study [31,36], which allowed a greater sample size per species (55 nests for *A. vigintipunctatum*, 274 nests for *M. leucographa*, 83 nests for *M. nigella*, 92 nests for *T. laticeps* and 51 for *X. atamisquensis*); because results of these estimations were qualitatively similar, we report here only results for the data of the two sites studied here.

### (e) Estimation of phenologies and nesting–flowering phenological mismatch

We used the weekly data on bee nest and flower abundance to estimate nesting and flowering phenologies. At the level of individual nests, we estimated phenology based on the end date of nest construction (i.e. the first date on which we observed that nest to be complete), which represented the most accurate date we could record in our weekly visits to the sites for three of our study species (*A. vigintipunctatum* and the two *Megachile* species), as females usually build the nests in less than a week. *Trichothurgus laticeps* appears to take slightly longer than a week, as we sometimes found nests under construction, while *X. atamisquensis* takes substantially longer (several weeks), but we decided to use also the end date of nest construction for these species to make data more comparable across species. At the population level, we estimated phenology using the abundance-weighted arithmetic mean date (WMD) of nesting or flowering, respectively, calculated with function weighted.mean of the stats package of R statistical software [37]. WMD is considered one of the best phenological estimators [38] and has been widely used in previous studies (e.g. [39–41]). Dates were represented as the number of days from 1 July each year, and the weighted average of nesting or flowering date was calculated by multiplying each date by the relative abundance of nests or flowers in that date. For example, if flower abundance was 10, 60 and 30 flowers on days 60, 67 and 74 from 1 July, the WMD of flowering is calculated as 0.1 × 60 + 0.6 × 67 + 0.3 × 74 = 68.4.

We used the above phenological estimates to quantify nesting–flowering phenological mismatches. For the analysis at the level of individual nests, we calculated phenological mismatch as the absolute difference between the end date of the nest construction and the flowering WMD of the main floral resource species (those that together represented 85% of the pollen found in all examined nests of a particular bee species). For the population-level analysis, we calculated the number of days between the nesting WMD and the flowering WMD to estimate the nesting–flowering phenological mismatch for each bee species and its main floral resource species.

Because we needed to record at least one nest to estimate the nesting–flowering phenological mismatch for a given year, we excluded from the analyses those years in which we recorded no nests of a particular species. Under the above definition of phenological mismatches, it is reasonable to expect mismatches both for specialist and generalist bee species, as in both cases the nesting and flowering dates are likely to shift among years [13,14].

### (f) Estimation of bee reproductive success and population growth rate

We estimated reproductive success for individual nests as the number of brood cells per nest, whereas at the population level we estimated reproductive success as the average number of brood cells per nest [42,43]. It should be noted that females of many bee species may provision multiple nests in succession [43,44], so that potential female fitness might be greater than cells per nest. For one of our study bee species, *X. atamisquensis*, we have observed females building only one nest during the entire nesting season, remaining to guard the nest once they finished building it. For the other four study species, the female leaves once she has finished building the nest, so we do not know the total number of brood cells produced by a female, as we could not follow individual females; thus, we cannot be certain whether only one nest is built by each female.

One species, *T. laticeps*, lacks organized brood cells, as females lay bare eggs amidst a pollen mass [35]. In turn, in some nests of *X. atamisquensis* adults emerged in the field before we could count the number of brood cells, and although some nests bore clear marks of the former brood cell divisions, in other nests the marks were less clear. Thus, for these species we used the length of the trap cavity occupied by pollen to estimate the number of brood cells in nests where a direct count was not possible. To this end, we first used a subset of nests for which we could count the number of pupal cocoons (*T. laticeps*) or brood cells (*X. atamisquensis*) to fit a linear regression model between number of brood cells or pupal cocoons and length of the cavity occupied by pollen (*T. laticeps*) or formerly occupied by brood cells (*X. atamisquensis*), and then used this relationship to estimate the number of brood cells in all nests from the length of the cavity occupied by pollen (see electronic supplementary material, methods for further information on the calculations).

We also calculated the average population growth rate, $\bar{\lambda }$, as the geometric mean of the population growth rate estimated for each study year, *λ_t_* = *N_t_*_+1_/*N_t_* [45], where *N_t_* and *N_t_*_+1_ are the total estimated number of brood cells produced in all nests of the population in years *t* and *t* + 1, respectively [42]. The geometric mean of *λ_t_* is then calculated as the arithmetic mean of log(*N_t_*_+1_/*N_t_*). Thus, when $\bar{\lambda }>0$ the population is expected to grow, whereas when $\bar{\lambda }<0$ the population is expected to decrease [45].

### (g) Statistical analyses

We used linear mixed models to assess the relationship between number of brood cells per nest and nesting–flowering phenological mismatch. We fitted two types of models separately for each bee species: linear and exponential. In both cases, we included year (the year in which flowering and nesting started in the spring) as a random variable in the model. Using the lme function in the nlme package of R statistical software [46], we ran linear models using as predictor variable the phenological mismatch in number of days and as response variable the number of brood cells per nest for the linear model or the natural logarithm of the number of brood cells for the exponential model. In both cases, we included year as a random factor. We then compared the fit of the two models using Akaike's Information Criterion. In all cases, the exponential model was the best fitting one, so we report this model in the main manuscript. In addition, to compare the coefficients of the effects of mismatch on number of brood cells among bee species, we standardized fixed predictor and response variables by subtracting the mean and dividing by the standard deviation, so as to obtain standardized regression coefficients, which allow comparison of the magnitude of coefficients. In addition, we used Spearman's correlation coefficient to evaluate the population-level relationship between bee reproductive success and phenological mismatch.

## 3. Results

We recorded a total of 766 bee nests, of which 433 belonged to *M. leucografa*, 151 to *A. vigintipunctatum*, 75 to *T. laticeps*, 62 to *X. atamisquensis* and 45 to *M. nigella*.

**Figure 1.**
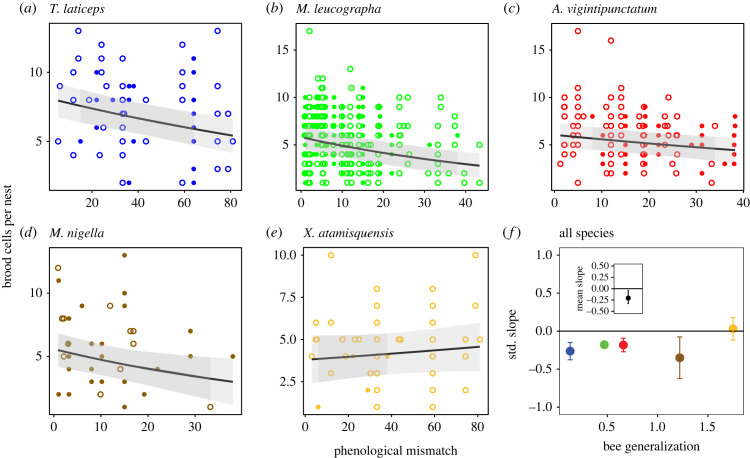
Phenological mismatch, bee specialization and nest-level reproductive success of solitary bees. (*a–e*) Relationship between bee reproductive success (number of brood cells per nest) and nesting–flowering phenological mismatch, from the most specialized (*a*) to the most generalized (*e*) bee species included in the study (see electronic supplementary material, tables S2 and S3 for model selection and fit statistics). Phenological mismatch was calculated as the absolute value of the difference between the nesting dates (end date of construction of each nest) and the weighted average of flowering dates (see Methods). Data were pooled for the two sites studied during 9 years. The line represents the fit from the linear model, and the shaded area above and below the line is the 95% confidence interval of the fitted line. Because field methodology was different for the first two study years (2006–2011), data for those years are shown with filled circles, while data for the remaining years (2008–2011) are shown with empty circles. (*f*) The strength of the relationship (standardized regression coefficient ± s.e.) in (*a*–*e*) versus bee generalization (Shannon diversity of pollen found in brood cells). Symbol colours in (*f*) match those in (*a–e*). The relationship is more strongly negative for the most specialized bee species, becoming weaker and non-significant for more generalized bee species. The inset at the top-left represents the mean ±95% bootstrap confidence limits of the standardized regression coefficient for all species.

For individual nests, the flowering–nesting phenological mismatch had a detectable statistical effect on reproductive success: the number of brood cells per nest was negatively related to the phenological mismatch with flowering phenology for all study species except the most generalized one, *X. atamisquensis* (figure 1*a–e*; electronic supplementary material, tables S2 and S3); furthermore, the slope of the relationship became weaker and non-significant with increasing bee generalization (figure 1*f*). The average standardized slope of this relationship across bee species was marginally significantly lower than zero (figure 1*f*). The number of brood cells per nest was also negatively related to nesting date (electronic supplementary material, figure S2a–e), although this effect was even weaker than for phenological mismatch, with the standardized slope of the relationship overlapping zero for three of the five study species (electronic supplementary material, figure S2f, tables S4 and S5) and the average standardized slope of this relationship across bee species also overlapping zero (electronic supplementary material, figure S2f).

**Figure 2.**
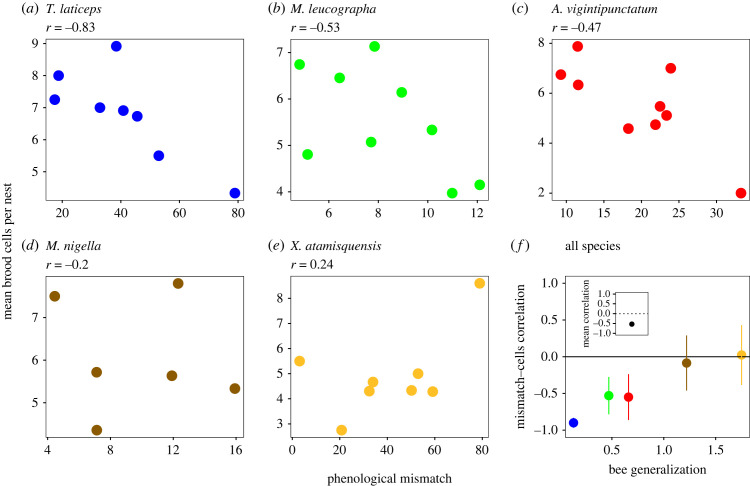
Phenological mismatch, bee specialization and population-level reproductive success of solitary bees. (*a–e*) Relationship between population-level bee reproductive success (yearly average of number of brood cells per nest) and nesting–flowering phenological mismatch, from the most specialized (*a*) to the most generalized (*e*) bee species included in the study. Phenological mismatch was calculated as the absolute value of the difference between the weighted averages of nesting and flowering dates (see Methods). Data were pooled for the two sites studied during 9 years. (*f*) The five coloured symbols on the right represent Spearman's correlation coefficient ± s.e. in (*a*–*e*) versus bee generalization (Shannon diversity of pollen found in brood cells); symbol colours match those in (*a–e*). The relationship is more strongly negative for the most specialized bee species, becoming weaker and non-significant for more generalized bee species. The inset at the top-left represents the mean ±95% bootstrap confidence limits of the correlation coefficient for all species.

**Figure 3.**
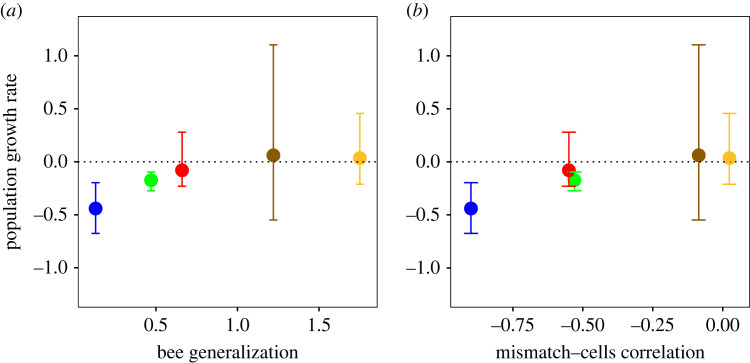
Geometric mean of population growth rate ($\bar{\lambda }$) of study bee species versus their (*a*) degree of specialization and (*b*) phenological mismatch. As in figures 1 and 2, data were pooled for the two sites studied during 9 years. Error bars represent the 95% percentile bootstrap confidence intervals of $\bar{\lambda }$, calculated by drawing with replacement 1000 bootstrap samples of the nests of each study bee species. Symbol colours indicate species identities as in figures 1 and 2. To calculate $\bar{\lambda }$, for a particular year *t* we defined the population growth rate as *λ_t_* = *N_t_*_+1_/*N_t_*, where *N_t_* and *N_t_*_+1_ are the total number of brood cells produced in all nests of the population in years *t* and *t* + 1, respectively [42]. The geometric mean of *λ_t_* is calculated as the arithmetic mean of log(*N_t_*_+1_/*N_t_*). Thus, when $\bar{\lambda }>0$ the population is expected to grow, whereas when $\bar{\lambda }<0$ the population is expected to decrease [45].

The above relationships between phenological mismatches and reproductive success at the level of individual nests are intriguing, but do not necessarily have an impact on population demography [26]. Therefore, next we address whether the above individual level effects of mismatches on reproductive success translate into a population-level effect on reproductive success and, ultimately, population growth rate. At the population level, the reproductive success of the three most specialized study bee species tended to decrease with increasing magnitude of phenological mismatches (figure 2). The average correlation coefficient across bee species was significantly lower than zero (figure 2*f*). Furthermore, the correlation became increasingly weaker with increasing bee generalization (figure 2*f*), which suggests that generalist bees were less affected by phenological mismatches than specialist bees. The decreasing reproductive effects of mismatches with increasing bee generalization were paralleled by a similar relationship between average population growth rate and bee generalization (figure 3), suggesting that phenological mismatches may put specialist bees at a greater risk of population decline than generalist bees.

## 4. Discussion

Our study provides evidence supporting the match/mismatch hypothesis for a group of solitary bees, showing that their individual- and population-level reproductive performance and population growth rate tend to decrease with increasing nesting–flowering mismatches, except for the most generalized bee species. Taken together, these results indicate that the combined weak but significant effects observed for individual nests lead to a stronger relationship between yearly population-level reproductive success and phenological mismatch. Thus, nesting too early or too late with respect to the flowering dates of the plant species on which bees rely has a detectable impact on the reproductive success of these solitary bees. Furthermore, the stronger negative effects of mismatches on reproductive success and the lower population growth rates estimated for the most specialized bee species suggest that specialists may suffer more from mismatches than generalists. Our findings add to a growing number of studies assessing whether phenological mismatches may disrupt species interactions [3–5] and influence the demography of interacting species [15–19,21].

Our finding of stronger demographic effects of mismatches for specialist than generalist bee species supports our expectations, but should be taken with caution, as our study focused on only five bee species, which precludes drawing strong conclusions about the influence of ecological specialization on species responses to phenological mismatches. Yet, these results make sense in light of the natural history of our study species. *Trichothurgus laticeps* uses almost exclusively the cactus *Opuntia sulphurea* as a source of pollen [35]. *Megachile leucographa* uses the shrubs *Larrea* spp. (mostly the dominant *Larrea divaricata* and possibly its less abundant congeners *L. cuneifolia* and *L. nitida*) as sources of pollen for brood cell provision and petal sources as brood cell building material [28]. *Anthidium vigintipunctatum*, although less specialized on its flower resources than *T. laticeps* and *M. leucographa*, relies heavily on pollen of the tree *Prosopis flexuosa* [47,48]. Thus, it seems unsurprising that phenological mismatches between the nesting of these species and the flowering of their floral resources leads to detectable effects on their demography. By contrast, the two most generalized species in our dataset, *M. nigella* and *X. atamisquensis*, rely on a much broader spectrum of species, which makes it less likely that some degree of mismatch with their main floral resources will affect their demography, as they may use alternative resources (see [28,30] and electronic supplementary material, figure S3).

Our demographic analysis indicates a negative population growth rate for the two most specialized bee species, and a greater, nonnegative growth rate for the other three species. This result suggests that specialist bees might have lower viability and face a greater risk of decline from phenological mismatches than generalists. We should interpret these results with caution, given the uncertainties of both the data and the analysis. Yet, the strength of this type of analysis lies in its usefulness as a tool to compare among populations and species. As Morris & Doak [45] have argued, while a particular numerical value of a viability measure may be difficult to interpret, the relative values for different populations may offer insights about the real differences in the viability of those populations. In this sense, our demographic analysis warns us of potential differences in the viability of our study bee species, presumably related to the greater impact of phenological mismatches on specialist species.

Our findings help elucidate the role of phenological mismatches for the demography of wild pollinators, which perform key ecosystem functions and provide important services for humanity [24,25]. Given the prospect of increased climatic variability expected in many of the world's regions [49], and the influence of climate on phenology and phenological mismatch [3–5,14,21,48], our results have important implications for our understanding of the potential ecological consequences of climate change.

## Data Availability

Data supporting this paper are available in Dryad Digital Repository: https://doi.org/10.5061/dryad.37pvmcvmp [50]. Supplementary figures, tables, and methods are provided in the electronic supplementary material [51].
